# Synthesis and Evaluation of Chalcone Derivatives as Novel Sunscreen Agent

**DOI:** 10.3390/molecules26092698

**Published:** 2021-05-04

**Authors:** Lucia Wiwid Wijayanti, Respati Tri Swasono, Wonkoo Lee, Jumina Jumina

**Affiliations:** 1 Department of Chemistry, Faculty of Mathematics and Natural Sciences, Universitas Gadjah Mada, Sekip Utara, Bulaksumur, Yogyakarta 55281, Indonesia; lwijayanti@usd.ac.id (L.W.W.); respati@ugm.ac.id (R.T.S.); 2 Chemistry Education Department, Kampus III Paingan, Universitas Sanata Dharma, Yogyakarta 55281, Indonesia; 3 Department of Chemistry, Sogang University, Seoul 04107, Korea; wonkoo@sogang.ac.kr

**Keywords:** chalcone, synthesis, UV-activity, cytotoxicity, sunscreen

## Abstract

Ultraviolet (UV) irradiation is a serious problem for skin health thus the interest in the research to develop sunscreen agent has been increasing. Chalcone is a promising compound to be developed as its chromophore absorbs in the UV region. Therefore, in the present work, we synthesized eight chalcone derivatives through Claisen–Schmidt condensation at room temperature. The evaluation of the optical properties of each chalcone derivatives in the UV region was conducted through spectroscopic and computational studies. The synthesized chalcones were obtained in good yields and they were active in the UV region. The results revealed that more methoxy substituents to chalcone leads toward red shift. All chalcone derivatives have high molar absorptivity value (21,000–56,000) demonstrating that they have the potential to be used as the sunscreen agent. The cytotoxicity assay showed that chalcone derivatives were demonstrating low toxicity toward normal human fibroblast cell, which is remarkable. Therefore, we concluded that the synthesized chalcones in this work were potential to be developed as novel sunscreen agents in real application.

## 1. Introduction

Sunlight provides many benefits for human health; however, sunlight can also generate negative effects. Several studies reported the adverse effects of ultraviolet (UV) irradiation in sunlight exposure. One of the oxidative stress indicators, the concentration of free radical in peripheral blood, was observed to be increasing in workers on Tuscany during the period of maximum solar UV irradiance [1]. The Scientific Committee on Emerging and Newly Identified Health Risks (SCENIHR) found that UV irradiation is a physical factor that initiates and promotes cancer disease [2]. Research on TP53 mutations provided strong evidence of a role for solar radiation in generating the etiology of squamous cell carcinoma of the conjunctiva [3]. UV irradiation is epidemiologically and molecularly linked to the three types of skin cancer, basal cell carcinoma, squamous cell carcinoma, and malignant melanoma diseases [4]. Another study reported an increment of reactive oxygen intermediates and inflammatory mediator due to UV-B light exposure inducing oxidative stress in primary cultures of mouse keratinocytes [5]. Mitochondrial damage of human dermal cells was also observed after exposure to artificial sunlight [6]. From in vitro and in vivo studies, UV–visible irradiation at boundary region (385–405 nm) significantly damaged skin cells and formed dark cyclobutene-pyrimidine dimers [7]. UV irradiation also plays a direct mutational role in promoting melanoma and oncogene induction as well as an indirect role through micro-environmental alterations in mouse [8]. Therefore, the protection of human skin from direct exposure to UV irradiation shall be carefully considered.

Sunscreen agents are critical products employed as photo-protectants against harm-ful UV rays. Higher peoples’ awareness of the risk of continuous exposure to the sun and its relation to cancer has increased the demand for effective sunscreen agents [9]. Clinical results on erythema and HbO_2_ content indicated that better sunburn protection could be achieved by using the SPF 100% sunscreen [10]. Following melanoma genetic assay on human population reported that daily routine usage of sun protection behavior within 2 years, decreased the number of sunburn cases [11]. Inorganic sunscreens are materials that scatter and reflect UV rays away from the protected area [9,12]. The most commonly used particulate sunscreens are titanium dioxide (TiO_2_) and zinc oxide (ZnO); however, their photo corrosion property makes their application is limited [13]. Meanwhile, plant extracts have been also evaluated as the subject of sunscreen studies [14,15,16,17,18,19]. However, their sunscreen activities are not satisfying due to the low concentration of the extracted compounds.

On the other hand, organic sunscreens generally consist of aromatic compounds linked with a carbonyl group. They are broadly classified into three categories based on the range of protection; UV-B (290–320 nm) and UV-A (320–400 nm), and broad-spectrum sunscreens that cover the entire spectrum (290–400 nm). The commonly used organic sunscreen agents are para-aminobenzoic acid (PABA), benzophenones, and ethylhexylmethoxycinnamate derivatives [20,21,22]. However, their efficacy as the sunscreen agent still needs to be improved.

**Scheme 1:**
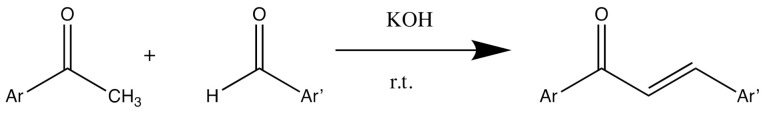
General reaction of chalcone synthesis.

It is reported that the chalcone derivatives absorb light in the UV region. The UV spectrum of chalcones consists of two essential absorption bands, i.e., band I and II as ma-jor and minor bands, respectively. Band I of chalcones usually appears at 220–270 nm, while band II appears at 340–390 nm. To be more specific, trans-Chalcones yield band I and II near 230 and 300 nm, in which the intensity of band II is much stronger than band I [23,24,25,26]. These bands are generated from n → π * and π → π * electronic transitions [25]. These electronic transitions offer a promising application as the sunscreen agent as chalcone derivatives can absorb the UV light before reaching the human skin. Furthermore, sunscreen agent based on chalcone derivatives is possible to develop for large-scale commercial production as chalcones can be easily synthesized by reacting benzaldehyde and acetophenone derivatives through a Claisen-Schmidt condensation reaction under the alkaline condition as shown in Scheme 1 [27,28,29,30,31,32,33,34,35].

## 2. Results and Discussion

### 2.1. Synthesis of Chalcone Derivatives

**Figure 1:**
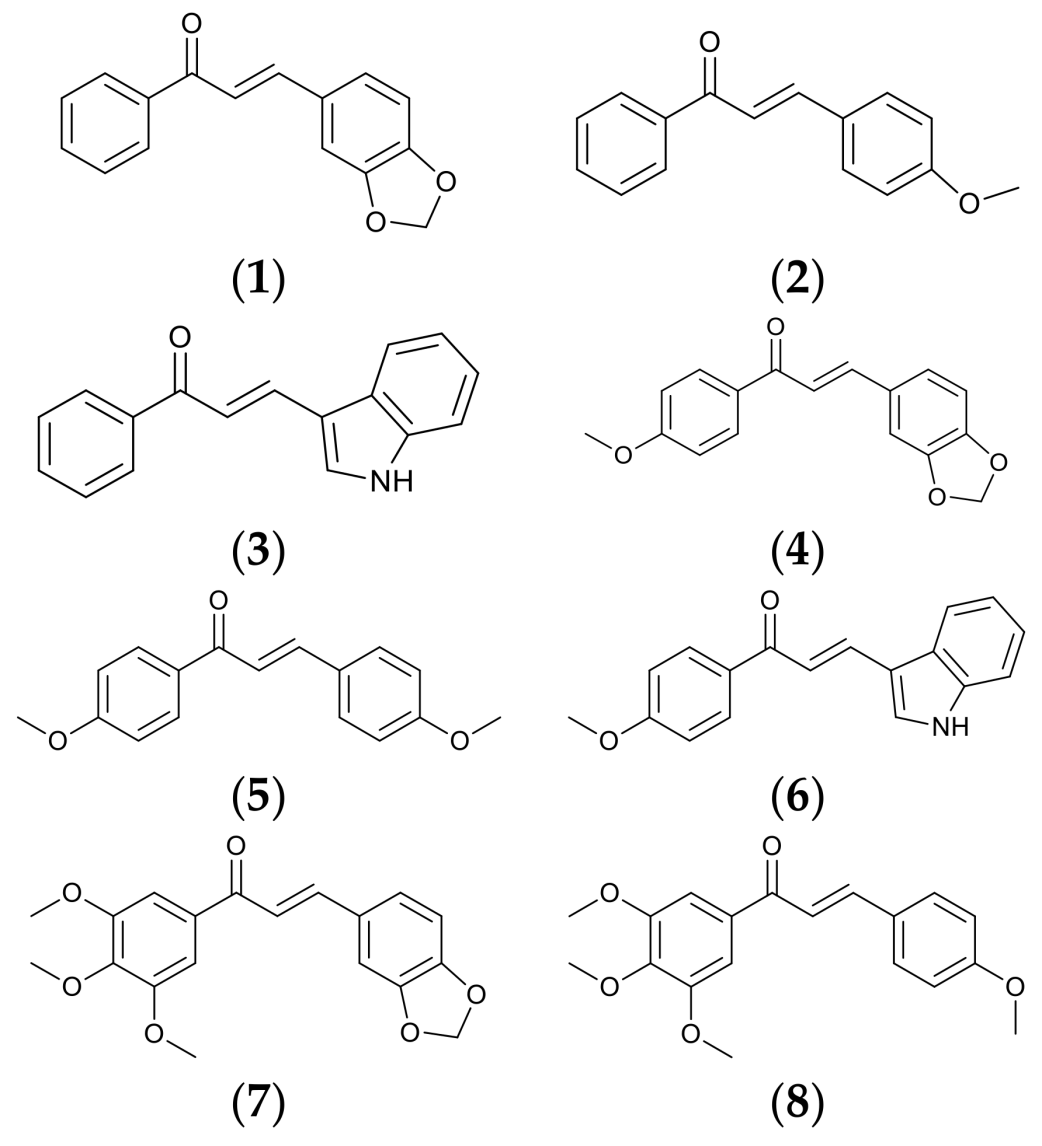
Chemical structure of chalcones **1**–**8**.

In this work, we synthesized eight chalcone derivatives and evaluated them as the sunscreen agent (Figure 1). In this research, the synthesis of chalcone derivatives was carried by using readily available starting materials, such as acetophenone, 4-methoxyacetophenone, 3,4,5-trimethoxyacetophenone, piperonal, 4-methoxybenzaldehyde (*p*-anisaldehyde), and 1-indole-3-carboxaldehyde (see Figure 1) in the one-pot reaction at room temperature, which in turn considered to be efficient reactions. The Claisen-Schmidt condensation reaction is a general organic reaction to obtain chalcone derivatives in medium to high yield. During this reaction, the hydroxide base attracts α-hydrogen from acetophenone derivatives to form enolate anions (carbanions). The carbanion attacks the carbonyl of benzaldehyde derivatives generating β-hydroxy carbonyl intermediates. This intermediate spontaneously produced chalcone under the acidic condition [26].

Because of that, the success of the reaction can be easily monitored from the disappearance of C–H aldehyde and the appearance of the C=C alkene functional group. As an example, the absorption signals at 2850 and 2750 cm^−1^ of C–H aldehyde were absent on the FTIR spectrum of compound **1**. On the other hand, an absorption signal at 984 cm^−1^ on the FTIR spectrum of chalcone **1** was found due to the presence of C=C alkene in trans geometry. From the ^1^H-NMR data, the C–H aldehyde signal at 9.79 ppm was absent in the ^1^H-NMR spectrum of compound **1** while the protons of C=C trans were found at 7.39 and 7.72 ppm as doublet signals with the coupling constant of 15.6 Hz. Furthermore, the ^13^C-NMR data of chalcone **1** shows carbon signals at 125.3 and 148.4 ppm for C=C trans confirming that chalcone **1** has been successfully synthesized. The other chalcone derivatives have been also obtained in 62.0–98.0% yield. Afterward, the synthesized compounds were evaluated through spectroscopic experiment as well as computational studies to know their potential application as sunscreen agents.

*(E)-3-(benzo[d][1,3]dioxol-5-yl)-1-phenylprop-2-en-1-one* (**1**)

Yield 98.0%. Yellow solid. FTIR: ν_max_/cm^−1^: 3030 (C–H sp^2^), 2922 (C–H sp^3^), 1658 (C=O), 1486 & 1559 (aromatic C=C), 1105 (C–O–C), 984 (C=C trans). ^1^H-NMR (CDCl_3_) δ/ppm: 6.04 (s, 2H, -OCH_2_O-), 6.86 (d, 1H, *J* = 8.00 Hz, Ar–H), 7.14 (d, 1H, *J* = 8.00 Hz, Ar–H), 7.18 (s, 1H, Ar–H), 7.39 (d, 1H, *J* = 15.2 Hz, C=C trans), 7.50 (t, 2H, *J* = 8.00 Hz, Ar–H), 7.58 (t, 1H, *J* = 8.00 Hz, Ar–H), 7.72 (d, 1H, *J* = 15.6 Hz, C=C trans), 7.99 (d, 2H, *J* = 8 Hz, Ar–H). ^13^C-NMR (CDCl_3_) δ/ppm: 101.6 (-OCH_2_O-), 125.3 & 148.4 (C=C trans), 190.4 (C=O), 106.6, 108.7, 120.1, 128.4, 128.6, 129.3, 132.7, 138.4 (C aromatics). Mass spectrum (ESI): *m*/*z* calcd. 252.26; found 253.08 [M^+^].

*(E)-3-(4-methoxyphenyl)-1-phenylprop-2-en-1-one* (**2**)

Yield 96.0%. Pale-yellow solid. FTIR: ν_max_/cm^−1^: 3010 (C–H sp^2^), 2955 (C–H sp^3^), 1684 (C=O), 1511 (aromatic C=C), 1171 (C–O–C), 984 (C=C trans). ^1^H-NMR (CDCl_3_) δ/ppm: 3.86 (s, 3H, -OCH_3_), 6.95 (d, 2H, *J* = 8.4 Hz, Ar–H), 7.40 (d, 1H, *J* = 15.6 Hz, C=C trans), 7.51 (t, 2H, *J* = 7.6 Hz, Ar–H), 7.58 (q, 3H, *J* = 8.4 Hz, Ar–H), 7.81 (d, 1H, *J* = 15.6 Hz, C=C trans), 8.02 (d, 2H, *J* = 7.6 Hz, Ar–H). ^13^C-NMR (CDCl_3_) δ/ppm: 55.61 (-OCH_3_), 123.9 & 144.9 (C=C trans), 190.8 (C=O), 113.8, 114.5, 119.9, 127.8, 128.6, 128.7, 129.0, 130.4, 131.8, 132.7, 138.7 (C aromatics). Mass spectrum (ESI): *m*/*z* calcd. 238.28; found 239.08 [M^+^].

*(E)-3-(1H-indol-3-yl)-1-phenylprop-2-en-1-one* (**3**)

Yield 62.0%. Light-brown solid. FTIR: ν_max_/cm^−1^: 3165 (NH), 3039 (C–H sp^2^), 1625 (C=O), 1516 & 1438 (aromatic C=C), 1105 (C–O–C), 994 (C=C trans). ^1^H-NMR (DMSO-d6) δ/ppm: 7.23 (m, 6H, -OCH_3_), 7.49 (d, 2H, *J* = 8.4 Hz, Ar–H), 8.09 (d, 2H, *J* = 8.4 Hz, Ar–H), 8.29 (s, 2H, Ar–H), 9.93 (s, 2H, Ar–H), 12.13 (s, 1H, NH). ^13^C-NMR (DMSO-d6) δ/ppm: 112.8 & 124.6 (C=C indole), 123.9 & 138.9 (C=C trans), 185.4 (C=O), 118.6, 121.2, 121.3, 122.6, 137.5 (C aromatics). Mass spectrum (ESI): *m*/*z* calcd. 247.29; found 248.16 [M^+^].

*(E)-3-(benzo[d][1,3]dioxol-5-yl)-1-(4-methoxyphenyl)prop-2-en-1-one* (**4**)

Yield 94.0%. Light-yellow solid. FTIR: ν_max_/cm^−1^: 3063 (C–H sp^2^), 2965 (C–H sp^3^), 1652 (C=O), 1581 & 1496 (aromatic C=C), 1235 (C–O–C), 992 (C=C trans). ^1^H-NMR (CDCl_3_) δ/ppm: 3.89 (s, 3H, -OCH_3_), 6.03 (s, 2H, *J* = 7.15 Hz, -OCH_2_O-), 6.86 (d, 1H, *J* = 8.00 Hz, Ar–H), 6.98 (t, 2H, *J* = 7.2 Hz, Ar–H), 7.12 (d, 1H, *J* = 6.8 Hz, Ar–H), 7.17 (s, 1H, Ar–H), 7.41 (d, 1H, *J* = 15.2 Hz, C=C trans), 7.75 (d, 1H, *J* = 15.2 Hz, C=C trans), 8.02 (m, 2H, Ar–H). ^13^C-NMR (CDCl_3_) δ/ppm: 55.63 (-OCH_3_), 101.7 (-OCH_2_O-), 125.9 & 148.2 (C=C trans), 187.3 (C=O), 106.9, 108.6, 114, 119.9 129.4, 130.6, 130.9, 143.3, 149.5, 163.2 (C aromatics). Mass spectrum (ESI): *m*/*z* calcd. 282.29; found 283.18 [M^+^].

*(E)-1,3-bis(4-methoxyphenyl)prop-2-en-1-one* (**5**)

Yield 95.0%. Pale-yellow solid. FTIR: ν_max_/cm^−1^: 3070 (C–H sp^2^), 2966 (C–H sp^3^), 1654 (C=O), 1591 & 1589 (aromatic C=C), 1165 (C–O–C), 985 (C=C trans). ^1^H-NMR (CDCl_3_) δ/ppm: 3.86 (s, 3H, -OCH_3_), 3.89 (s, 3H, -OCH_3_), 6.97 (m, 4H, Ar–H), 7.45 (d, 1H, *J* = 15.6 Hz, C=C trans), 7.61 (d, 2H, *J* = 8.8 Hz, Ar–H), 7.85 (d, 1H, *J* = 15.6 Hz, C=C trans), 8.05 (d, 2H, *J* = 7.6 Hz, Ar–H). ^13^C-NMR (CDCl_3_) δ/ppm: 55.37 & 55.46 (-OCH_3_), 127.7 & 143.7 (C=C trans), 188.7 (C=O), 113.7, 114.3, 119.5, 130.1, 130.7, 131.3, 161.5, 163.2 (C aromatics). Mass spectrum (ESI): *m*/*z* calcd. 268.31; found 269.16 [M^+^].

*(E)-3-(1H-indol-3-yl)-1-(4-methoxyphenyl)prop-2-en-1-one* (**6**)

Yield 65.0%. Yellow solid. FTIR: ν_max_/cm^−1^: 3165 (NH), 3040 (C–H sp^2^), 2928 (C–H sp^3^), 1628 (C=O), 1519 (aromatic C=C), 1119 (C–O–C), 982 (C=C trans). ^1^H-NMR (DMSO) δ/ppm: 2.45 (s, 3H, -OCH_3_), 6.52 (d, 3H, *J* = 8.00 Hz, Ar–H), 7.23 (m, 6H), 8.09 (d, 2H, *J* = 7.24 Hz), 8.28 (s, 3H, Ar–H), 9.93 (s, 3H, Ar–H). ^13^C-NMR (DMSO) δ/ppm: 30.73 (-OCH_3_), 118.2 & 124.1 (C=C indole), 123.5 & 138.5 (C=C trans), 185.0 (C=O), 120.8, 122.2, 137.1 (C aromatics). Mass spectrum (ESI): *m*/*z* calcd. 277.32; found 279.16 [M^+^].

*(E)-3-(benzo[d][1,3]dioxol-5-yl)-1-(3,4,5-trimethoxyphenyl)prop-2-en-1-one* (**7**)

Yield 98.0%. Yellow solid. FTIR: ν_max_/cm^−1^: 3012 (C–H sp^2^), 2934 (C–H sp^3^), 1654 (C=O), 1574 & 1491 (aromatic C=C), 1118 (C–O–C), 991 (C=C trans). ^1^H-NMR (CDCl_3_) δ/ppm: 3.93 (m, 12H, -OCH_3_), 6.04 (s, 2H, -OCH_2_-), 6.87 (d, 1H, *J* = 8.00 Hz, Ar–H), 7.15 (d, 1H, *J* = 8.00 Hz), 7.18 (s, 1H, Ar–H), 7.30 (d, 1H, *J* = 15.2 Hz, C=C trans), 7.77 (d, 1H, *J* = 15.2 Hz, C=C trans). ^13^C-NMR (CDCl_3_) δ/ppm: 56.34 & 60.96 (-OCH_3_), 101.6 (-OCH_2_O-) 125.3 & 148.4 (C=C trans), 189.1 (C=O), 105.9, 106.6, 108.7, 119.65, 129.3, 133.7, 142.3, 144.6, 149.9, 153.1 (C aromatics). Mass spectrum (ESI): *m*/*z* calcd. 342.34; found 343.16 [M^+^].

*(E)-3-(4-methoxyphenyl)-1-(3,4,5-trimethoxyphenyl)prop-2-en-1-one* (**8**)

Yield 71.0%. Pale-yellow solid. FTIR: ν_max_/cm^−1^: 3006 (C–H sp^2^), 2942 (C–H sp^3^), 1654 (C=O), 1568 & 1506 (aromatic C=C), 1119 (C–O–C), 978 (C=C trans). ^1^H-NMR (CDCl_3_) δ/ppm: 3.86 (s, 3H, -OCH_3_), 3.94 (s, 3H, -OCH_3_), 3.95 (s, 6H, -OCH_3_), 6.96 (d, 2H, *J* = 8.80 Hz, Ar–H), 7.25 (d, 2H, Ar–H), 7.38 (d, 2H, *J* = 15.6 Hz, C=C trans), 7.63 (d, 2H, *J* = 8.80 Hz, Ar–H), 7.82 (d, 1H *J* = 15.2 Hz, C=C trans). ^13^C-NMR (CDCl_3_) δ/ppm: 55.24, 56.17 & 60.79 (-OCH3), 127.4 & 142. (C=C trans), 189.1 (C=O), 105.5, 105.7, 108.7, 114.2, 119.2, 130.5, 133.6, 144.5, 152.9, 161.5 (C aromatics). Mass spectrum (ESI): *m*/*z* calcd. 328.4; found 329.25 [M^+^].

### 2.2. UV Absorbance Profile of Chalcone Derivatives

UV spectroscopy measurement of chalcone derivatives **1**–**8** was conducted in order to characterize their optical properties in the UV region (200–400 nm), such as cut off wavelength (λ_cut off_), maximum wavelength (λ_max_), and molar extinction coefficient (ε). Table 1 also lists the λ_cut off_ of chalcone derivatives **1**–**8**. The result of λ_cut off_ measurement showed that the empirical λ_cut off_ was obtained in a smaller wavelength region in comparison to that of theoretical prediction. This difference is possibly attributed to solvent interference in the experimental and computational studies.

**Figure 2:**
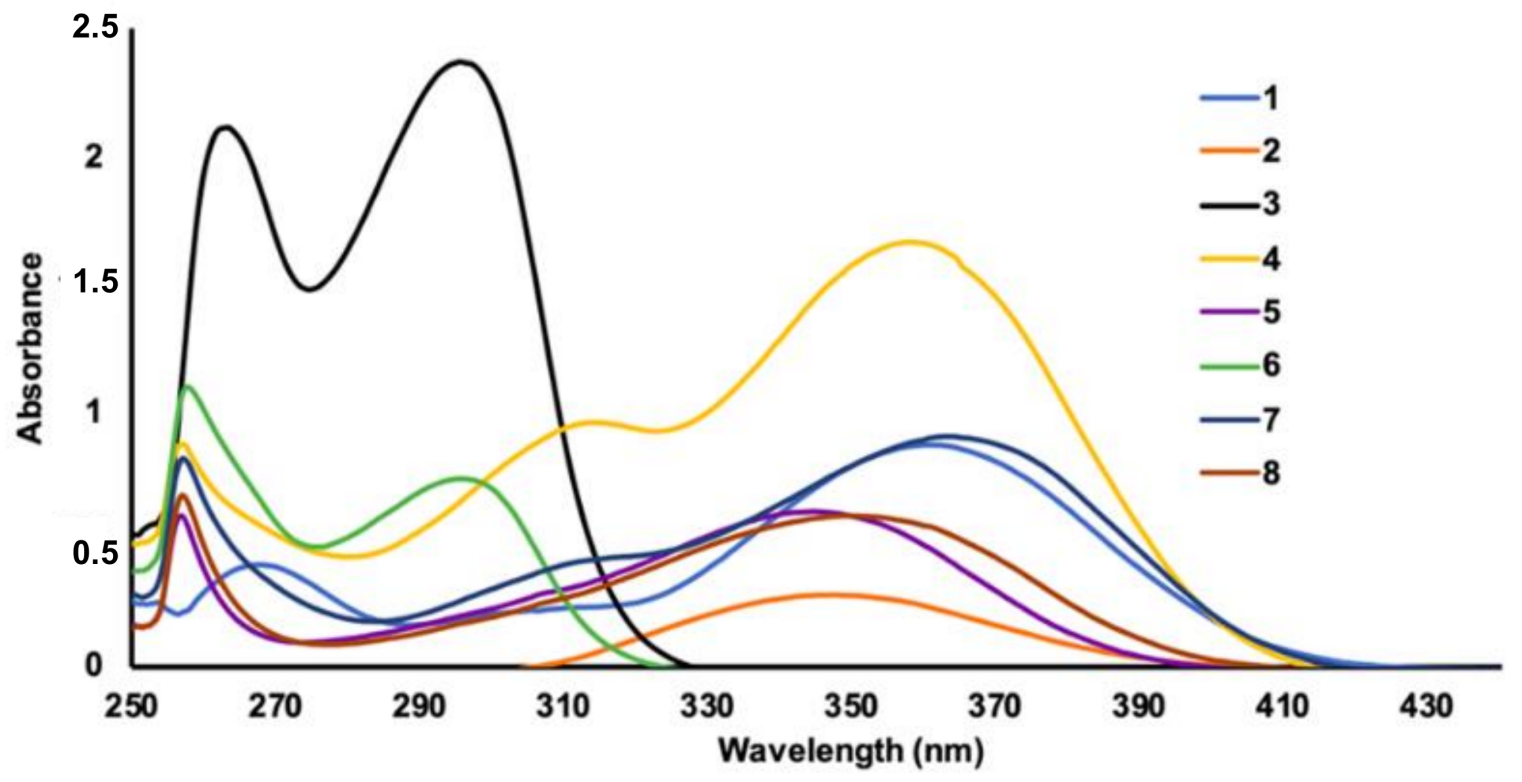
Experimental UV–vis spectra of synthesized chalcone derivatives **1**–**8**.

**Figure 3:**
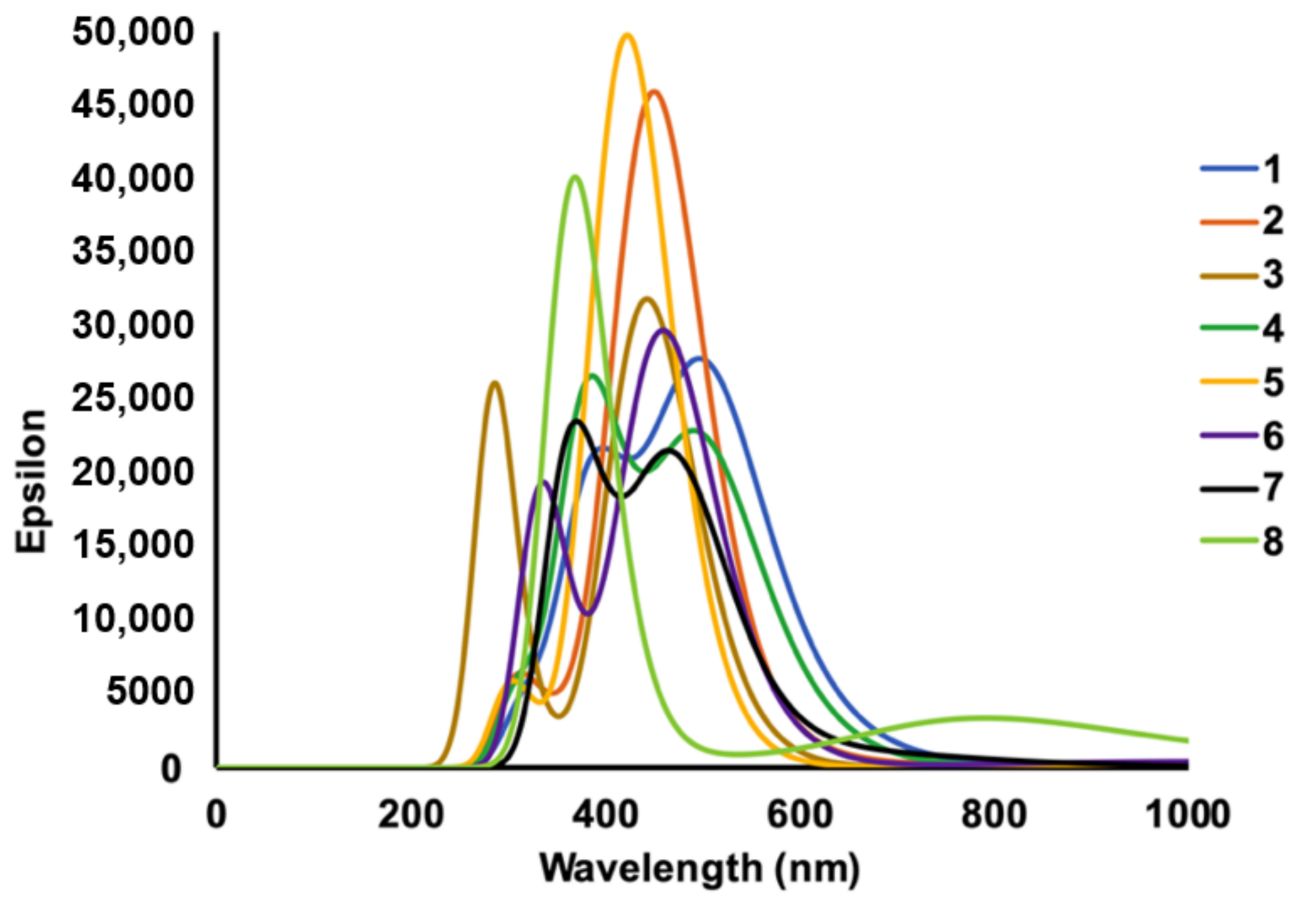
Theoretical UV–vis spectra of synthesized chalcone derivatives **1**–**8** from the computational study.

Figure 2 shows that more methoxy substitutions to the chalcones lead to the shift toward higher wavelength. These results suggested that the presence of the methoxy group as an electron-donating group stabilized the electron delocalization in the chalcone structure. This phenomenon in experimental is consistent with the results of computational study (see Figure 3 and Table 1).

The ε value of chalcone derivatives **1**–**8** was obtained in high molar absorptivity value (21,000–56,000). Again, this fact is consistent with the result of the predicted molar extinction coefficient using theoretical computation which showed that the molar extinction coefficient of chalcone derivatives **1**–**8** was obtained in the range of 20,000–48,000. This phenomenon is possibly attributed to π–π * excitation thus that chalcone derivatives **1**–**8** gave high absorptivity in the UV region. It is promising that chalcone derivatives **1**–**8** have the potential to be used as sunscreen agent. The λ_max_ and ε values of compound **2** (Table 1 in DMSO) is different from the previously reported (λ_max_ = 340 nm, ε = 19,000 in CHCl_3_) due to different solvent media or as known as the solvatochromic effect [36].

### 2.3. Cytotoxicity Assay

**Figure 4:**
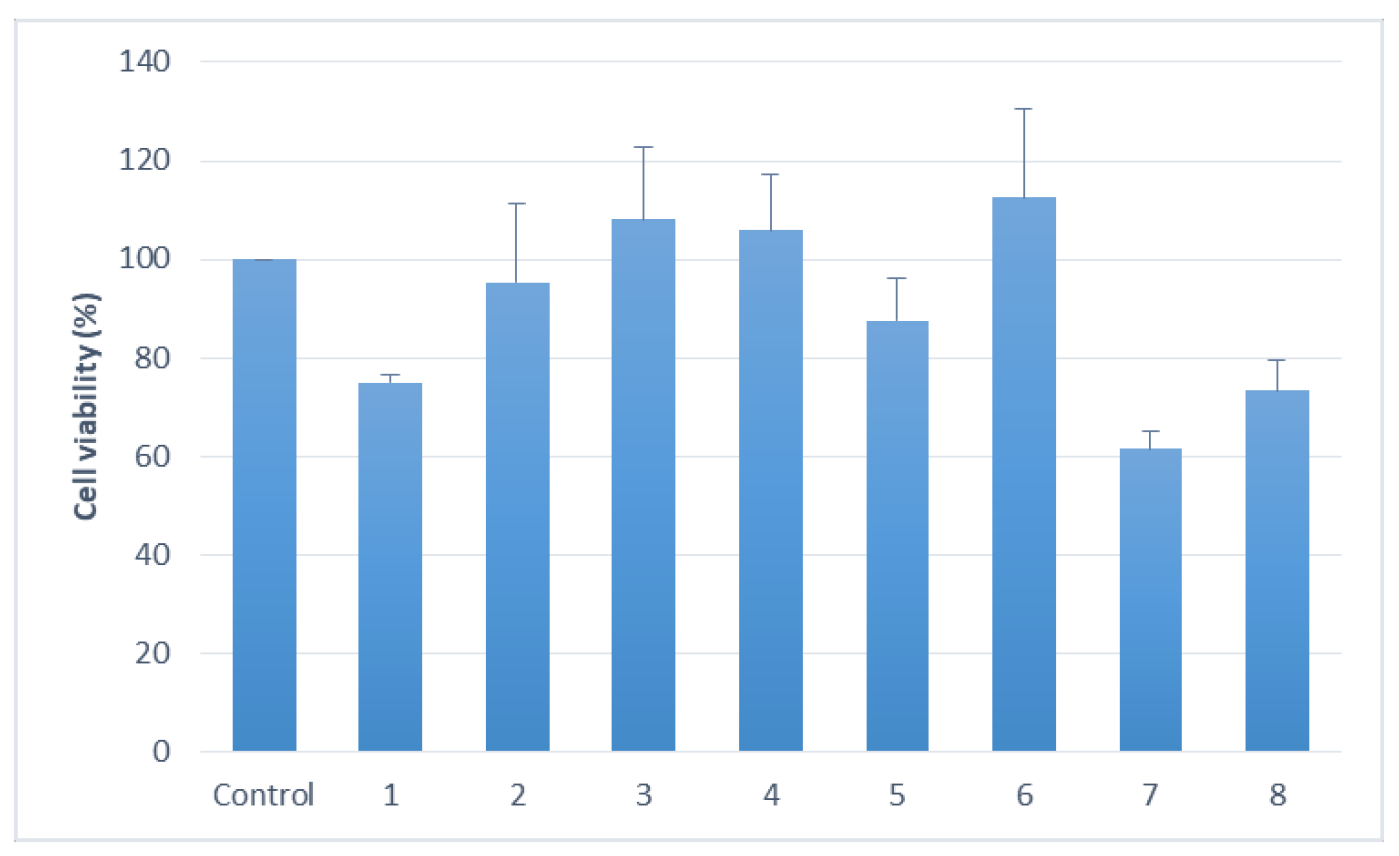
Cell viability (%) upon treatment with chalcone (**1**–**8**) in concentration of 50 ppm.

Figure 4 shows the cytotoxicity assay of a series chalcone **1**–**8**. Cytotoxicity was tested by WST assay toward normal human fibroblast cells (HDFn). In the WST assay, the quantity of formazan (presumably directly proportional to the number of viable cells) is measured by recording changes in absorbance using a plate reading spectrophotometer. Viable cells with active metabolism convert tetrazolium compound into purple-colored formazan product. When cells died, they lose the ability to convert tetrazolium compound into formazan, thus color formation serves as a useful and convenient marker of only the viable cells. WST reagents were reported as one of the more recently developed tetrazolium reagents that can be reduced by viable cells to generate formazan products that are directly soluble in cell culture medium. These improved tetrazolium reagents eliminate a liquid handling step during the assay procedure because the second addition of reagent to the assay plate is not needed to solubilize formazan precipitates, thus making the protocols more convenient [37].

Cell viability was expressed as percentage of the control. Values are mean ± standard deviation (SD) of three separate experiments, with three wells each. The result showed that the percentages of living human fibroblast cell were higher than 50% under treatment of 50 ppm of chalcones. Chalcones **2**, **3**, **4**, and **6** are the most promising candidates (about 100% of the percentage of living cells) from the cytotoxicity assay. All compounds **2**, **3**, **4**, and **6** have one methoxy functional groups showing that chalcones having one methoxy group are not toxic for human fibroblast cells.

## 3. Materials and Methods

### 3.1. Materials

Chemical reagents in this work are KOH, acetophenone, 4-methoxyacetophenone, 3,4,5-trimethoxyacetophenone, piperonal (3,4-(methylenedioxy)benzaldehyde), 4-methoxybenzaldehyde (*p*-anisaldehyde), 1-indole-3-carboxaldehyde, dimethyl sulfoxide (DMSO), ethanol, methanol, dichloromethane, ethyl acetate, and *n*-hexane. All reagents and solvents were commercially available in pro analytical grade.

For the cytotoxicity assay, the cells and reagents used were Human Neonatal Dermal Fibroblasts (HDFn, PCS-201-010, American Type Culture Collection (ATCC), Manassas, VA, USA), WST reagent EZ-CYTOX (from DoGenBio Co., Ltd., Seoul, Republic of Korea), 96-well plate, Dulbecco’s modified Eagle’s medium (DMEM), Fetal Bovine Serum (FBS), and antibiotics (penicillin and streptomycin).

### 3.2. Instruments Analysis

The ^1^H- and ^13^C-NMR spectra of synthesized compounds were recorded using NMR spectroscopy (Varian-NMR VNMRS-400) 400 MHz (^1^H) and 100 MHz (^13^C) spectrometers) in CDCl_3_ and DMSO-d6 with TMS as the internal standard. FTIR spectra of synthesized compounds were recorded using an FTIR spectrophotometer Shimadzu Spirit. The purity and mass spectra of the synthesized products were characterized by liquid-chromatography-mass spectrometer (ESI-Iontrap Mass Spectrometer LTQ XL model, Thermo Fischer Scientific, San Jose, CA, USA). Reaction and purity test were monitored through a thin-layer chromatography (TLC) on aluminum sheets silica gel 60 F254 plates (Merck) with an UV lamp (Camag, Muttenz, Switzerland) at 254 nm as a detecting unit (eluent: *n*-hexane/ethyl acetate in 2:4 ratio). The UV spectra were measured by double beam UV–vis spectrophotometer Shimadzu UV 1800 (Shimadzu Corp., Kyoto, Japan).

### 3.3. General Procedure Synthesis of Chalcone Derivatives

The cold solution of 0.893 g KOH (0.01 mol) in 6.5 mL of distilled water was added dropwise into a solution of 3.65 mmol of acetophenone derivatives in 3 mL of ethanol. A total of 3.65 mmol of benzaldehyde derivatives in 3 mL of ethanol was then introduced into the mixture and the mixture was stirred at room temperature. The mixture was then cooled in an ice bath. The formed solid product was filtered, recrystallized from methanol, and dried. The purity and product characterizations were performed using TLC (dichloromethane:*n*-hexane = 4:1), FTIR spectrometry, MS, ^1^H-, and ^13^C-NMR.

### 3.4. Determination of the UV–Vis Absorbance Profile of Chalcone Derivatives

The UV–vis spectroscopy measurement was recorded using a UV–vis spectrophotometer to obtain the absorbance profile including λ_cut off_, λ_max_, and ɛ values. The optimization of concentration was conducted by preparing three concentrations of each synthesized compound in 25 × 10^0^, 25 × 10^−2^, and 25 × 10^−4^ mM. The UV–vis spectra of these three samples of each synthesized compound were recorded in the range of 200–400 nm using a 1 cm quartz cuvette with DMSO as the blank solution. The concentration that produced a smooth line curve was selected for further measurement and calculation of λ_cut off_, λ_max_, and ɛ values. The ɛ value was determined by Equation (1).

A = l × ɛ × c(1)

where, A= absorbance, l = length of cuvette, ɛ = molar absorptivity (M^−1^ cm^−1^), c = concentration (M).

### 3.5. Computational Study

For UV spectrum analysis, Gaussian09 was chosen to perform a geometry optimization of chalcones molecule with time dependent-density functional theory (TD-DFT) method for accurate calculation of ground and excitation state. This method was supported with 6-31G(d,p)/PBEPBE basis set, additional orbital (d,p) for extra polarization function to increase λ_max_ by 1. Microsolvation was performed with chalcones and DMSO as solute and solvent, respectively. The solvent effect played an important role in spectra absorption of chalcone, so the type of solvent is crucial. Polarizable continuum model (PCM) was chosen to deal with the solvent effect where solvent makes a cavity for the solute to take the position. The bandgap, HOMO and LUMO energies were determined to predict chalcones conductivity.

### 3.6. Cytotoxicity Assay

Primary human dermal fibroblast neonatal (HDFn) cells were grown in DMEM containing 10% FBS and 1% antibiotics (penicillin and streptomycin). The cells were incubated in a humidified (95% air and 5% CO_2_) incubator at 37 °C. Cells were cultured to the confluence of 80–90% then seeded into a 96-well plate. After 24 h, the cells attached to the bottom of the wells and treated with the chalcone derivative. The WST reagent diluted with DMEM were added after the 24 h of treatment. After additional 2 h, the absorbance was measured at 450 nm. Each assay contained control and solvent control. The control contained cells in media and the solvent control contained the volume of solvent used. Cell viability was expressed as percentage of the control. Values are mean ± standard deviation (SD) of three separate experiments, with three wells each. The method design is an adaptation from previously reported studies [38,39].

## 4. Conclusions

The synthesis of eight chalcone derivatives has been successfully conducted at room temperature in 62.0–98.0% yields. All compounds were found to be active in the UV region. Chalcone derivatives with and without methoxy functional group absorb light at the UV region, in which more methoxy substituents leads to the red shift. All chalcone derivatives have high molar absorptivity value (21,000–56,000) demonstrating that they have the potential to be used as sunscreen agent. The cytotoxicity evaluation showed that chalcone derivatives have low toxicity toward normal human fibroblast cell, which is remarkable. In conclusion, the synthesized compounds have potential to be developed as novel sunscreen agents.

## Figures and Tables

**Table 1:** Comparison of empirical experimental and theoretical data of UV–vis absorbance profile of chalcones **1**–**8**.

Chalcone	Experimental			Theoretical		
	λ_max_ (nm)	ε (L mol^−1^ cm^−1^)	λ_cut off_ (nm)	λ_max_ (nm)	ε (L mol^−1^ cm^−1^)	λ_cut off_ (nm)
**1**	269	34,720	-	405	27,627	277
	363		426	506		866
**2**	-	56,400	306	221	45,473	192
	352		406	276		397
**3**	265	47,500	-	209	31,282	160
	299		326	323		473
**4**	258	33,840	-	235	25,753	211
	363		415	253		302
**5**	258	46,400	-	166	49,405	152
	349		398	190		241
**6**	259	21,400	-	171	29,139	156
	299		324	196		262
**7**	258	21,600	-	199	23,529	185
	367		418	213		286
**8**	257	32,080	-	183	38,716	153
	355		405	319		497

## Data Availability

The data presented in this study are available on request from the corresponding author.

## Sample Availability

Samples of all compounds are available from the authors upon the reasonable request.
